# Imaging the effect of receptor for advanced glycation endproducts on angiogenic response to hindlimb ischemia in diabetes

**DOI:** 10.1186/2191-219X-1-3

**Published:** 2011-06-07

**Authors:** Yared Tekabe, Xiaoping Shen, Joane Luma, Drew Weisenberger, Shi Fang Yan, Roland Haubner, Ann Marie Schmidt, Lynne Johnson

**Affiliations:** 1 Department of Medicine, Columbia University Medical Center, New York, NY 10032, USA; 2 Department of Surgery, Columbia University Medical Center, New York, NY 10032, USA; 3 Department of Medicine, New York University Medical Center, New York, NY 10032, USA; 4 Thomas Jefferson National Accelerator Facility, Newport News, VA 23606, USA; 5 Department of Nuclear Medicine, Medical University of Innsbruck, Innsbruck, Austria

## Abstract

**Background:**

Receptor for advanced glycation endproducts (RAGE) expression contributes to the impaired angiogenic response to limb ischemia in diabetes. The aim of this study was to detect the effect of increased expression of RAGE on the angiogenic response to limb ischemia in diabetes by targeting α_v_β_3 _integrin with ^99m^Tc-labeled Arg-Gly-Asp (RGD).

**Methods:**

Male wild-type (WT) C57BL/6 mice were either made diabetic or left as control for 2 months when they underwent femoral artery ligation. Four groups were studied at days 3 to 7 after ligation: WT without diabetes (NDM) (*n *= 14), WT with diabetes (DM) (*n *= 14), RAGE^-/- ^NDM (*n *= 16), and RAGE^-/- ^DM (*n *= 14). Mice were injected with ^99m^Tc-HYNIC-RGD and imaged. Count ratios for ischemic/non-ischemic limbs were measured. Muscle was stained for RAGE, α_v_β_3_, and lectins.

**Results:**

There was no difference in count ratio between RAGE^-/- ^and WT NDM groups. Mean count ratio was lower for WT DM (1.38 ± 0.26) vs. WT NDM (1.91 ± 0.34) (*P*<0.001). Mean count ratio was lower for the RAGE^-/- ^DM group than for RAGE^-/- ^NDM group (1.75 ± 0.22 vs. 2.02 ± 0.29) (*P*<0.001) and higher than for the WT DM group (*P*<0.001). Immunohistopathology supported the scan findings.

**Conclusions:**

*In vivo *imaging of α_v_β_3 _integrin can detect the effect of RAGE on the angiogenic response to limb ischemia in diabetes.

## Background

The prevalence of peripheral artery disease in the general population is 12% to 14%, affecting 20% of those >70 years and contributes to significant morbidity. Limb ischemia in diabetics takes a particularly malignant course leading to impaired wound healing, gangrene, amputations, and even death [1,2]. A major and distinct adaptive process that contributes to restoring nutrient blood flow to ischemic limbs is angiogenesis/arteriogenesis. Angiogenesis refers to the process of endothelial sprouting. Arteriogenesis is the formation of larger "arteriole" like vessels. Both processes are essential for the development of subsequent collateral growth [3]. Tissue hypoxia activates genes that code for angiogenic growth factors and cytokines. Investigational studies have documented the involvement of receptor for advanced glycation endproducts (RAGE) in the impaired angiogenic response to limb ischemia in diabetes [4-7].

The expression of α_v_β_3 _integrin, a cell adhesion receptor that plays a crucial role in the angiogenesis process, can be targeted with radiolabeled peptides for *in vivo *imaging [8]. Comparing *in vivo *imaging in animals with genetic alteration of pathways implicated in angiogenesis allows exploration of downstream effects in live animals. In this study, we investigated the value of imaging the effects of RAGE expression on the angiogenic response to limb ischemia in live animals. We used ^99m^Tc-labeled Arg-Gly-Asp (RGD) peptide that targets α_v_β_3 _integrin expression occurring during capillary sprouting. Our hypothesis was that using genetically altered mice, ^99m^Tc-labeled RGD imaging can detect *in vivo *the effect of RAGE expression on angiogenic response to limb ischemia in diabetes.

## Methods

### Experimental protocol

All animal experiments were performed in accordance with the approval of the Institutional Animal Care and Use Committee of Columbia University. Homozygous male RAGE null (RAGE^-/-^) mice (backcrossed >10 generations into C57BL/6) were generated as described previously [9]. Male wild-type (WT) C57BL/6 mice were obtained (Jackson Laboratories). At age 6 weeks, half of the WT and half of the RAGE^-/- ^mice were treated with streptozotocin (STZ; Sigma). Two months later, all mice underwent femoral artery (FA) ligation.

### Induction of diabetes

Mice were treated with five consecutive daily doses of STZ dissolved in citrate buffer (55 mg/kg, pH 4.5) via the intraperitoneal route. One week after the first dose, glucose levels were assessed by glucometer. The criteria of two consecutive glucose levels >250 mg/dL was used to indicate diabetes. If glucose levels were <250 mg/dL, then the mice received two additional doses of STZ (55 mg/kg).

### Femoral artery ligation

Under isoflurane anesthesia, the hair on the abdominal wall and pelvis and both upper legs was shaved and the skin prepped with iodine and alcohol. An incision was made on the upper thigh of both the left and right legs of each mouse. The inguinal ligament and the upper half of the femoral artery were exposed. On the left side, the vascular bundle was isolated from below the inguinal ligament proximally to just above the bifurcation into the superficial and deep femoral arteries distally. The femoral artery was dissected free, and two ligatures were placed around it with 8/0 non-absorbable sutures and tied. Both skin incisions were closed with sterile 5/0 nylon suture.

### Preparation of radiotracer

Aliquots of 5 μg of HYNIC-RGD were incubated with 0.5 ml of tricine solution (70 mg/ml in distilled water) and approximately 0.5 ml of ^99m^TcO_4_^- ^solution (50 mCi = 1,850 MBq) and 20 μl of tin(II) solution (10 mg of SnCl_2_·2H_2_O in 10 ml of nitrogen-purged 0.1 N HCl for 20 min) at room temperature. To test the specificity of the HYNIC-RGD, cyclo [Arg-Ala-Asp-D-Phe-Lys (HYNIC)] (Peptides International, Louisville, KY, USA) was similarly radiolabeled and used as control peptide. Radiochemical purity was >94% by Tec-control chromatography (Biodex, Shirley, NY, USA).

### Injection of radiotracer and imaging

Under isoflurane anesthesia (1.5% isoflurane at a flow rate of 0.5% L/min oxygen per mouse), a cutdown was made over the jugular vein and a specially designed vascular catheter was placed (Braintree Scientific, Braintree, MA, USA). Mice in each of four groups were injected with ^99m^Tc-HYNIC-RGD and imaged 3 or 7 days after FA ligation: WT without diabetes (*n *= 14), WT with diabetes (*n *= 14), RAGE^-/- ^without diabetes (*n *= 16), RAGE^-/- ^with diabetes (*n *= 14), and five WT without diabetes were injected with control peptide. All mice were injected through the jugular vein catheter with 1 ± 0.2 mCi of ^99m^Tc-HYNIC-RGD in 0.05 to 0.1 ml (corresponding to 1 μg of peptide) or control peptide. Blood pool clearance was measured in five mice injected with ^99m^Tc-HYNIC-RGD. By 60 to 75 min after injection, residual blood pool activity was below 10% of peak. Whole-body planar gamma images in the anteroposterior view were acquired on a high-resolution high-sensitivity dedicated small animal camera with parallel hole collimator (provided by Jefferson Lab, Newport News, VA, USA). The camera is based on a 5-in. Hamamatsu position sensitive photomultiplier type R3292 with an active field of view of about 95 mm diameter. The scintillator sensor is 1.6-mm-step 6-mm-thick pixelated NaI(Tl) scintillator array. The photo peak was set at 140 keV with a 15% energy window.

### *Ex vivo *tissue counting

At completion of the imaging session, each animal was euthanized by an intraperitoneal injection of pentobarbital (100 mg/kg). The anterior tibialis muscles were dissected, weighed, and counted in a gamma counter (Wallac Wizard 1470, PerkinElmer, Waltham, MA, USA) for determination of the percent injected dose of radiotracer per gram (%ID/g) tissue. The radiotracer activity in the samples was corrected for background, decay time, and tissue weight. Limb counting was performed in 28 animals. The remaining animals were used for immunohistochemistry.

### Histopathology

For immunohistochemical analyses, tibialis anterior muscles were harvested and fixed in 10% formalin for 48 h. Specimens were embedded in paraffin, and tissue slices (5 μm in thickness) were prepared. Serial sections were stained with hematoxylin and eosin (H&E) for morphology. Immunostaining was performed for capillary sprouting using biotinylated Griffonia Bandeiraea Simplicifolia Isolectin I (Vector Laboratories, Burlingame, CA, USA) for β_3 _(1:50; Abcam, Cambridge, MA, USA.) and for α_ν _(1:100; Millipore, Temecula, CA, USA). Serial sections were also stained for RAGE using a monoclonal antibody against RAGE (50 μg/ml). Secondary stains were performed using avidin-biotin visualization systems (Vectastain ABC Kit, Vector Laboratories). All brown staining capillaries were counted for each of 5 to 6 sections for both the left and right anterior tibialis muscles for each experiment and then were averaged. The average number of capillaries for the left anterior tibialis muscle was divided by the average number for the right (control) anterior tibialis muscle. RAGE staining was quantified as area staining positive for the brown chromagen per 100× field.

### Immunofluorescence

Dual immunofluorescent studies were undertaken to determine the cell types expressing α_ν _integrin. Serial sections (5 μm in thickness) obtained from the ischemic hindlimb were deparaffinized in xylene and incubated with α_ν _(rat anti-mouse integrin α_ν_, 1:100) and co-stained with endothelial cell marker (FVIII, 1:200) or macrophage marker (Mac-3, 1:50). Secondary fluorescent antibodies were Texas Red anti-rabbit and FITC anti-mouse. The images were captured and processed using confocal fluorescence microscope (Nikon, Tokyo, Japan) and SPOT imaging software (Diagnostic Instruments, Inc., Sterling Heights, MI, USA).

### Image analysis

Radiotracer counts in the ischemic hindlimb were determined from the *in vivo *scans by using the region of interest (ROI) method in the mini gamma camera image using public domain Image J software (NIH, Bethesda, MD, USA). A region was drawn around the focal uptake, and the mean counts were determined. Radioactivity in the contralateral control limb was similarly determined using a comparable ROI (same anatomic location and the number of pixels). The counts from each of these areas were used to determine the ischemic to non-ischemic ratios.

### Statistical analysis

Continuous variables were expressed as mean ± standard deviation. Normality was assessed using the Shapiro-Wilk test. Comparisons between two groups were made using the Student's *t *test. Correlation was assessed using the Pearson product-moment correlation coefficient. All statistical tests were two-tailed, with *P *< 0.05 denoting significance. All statistical analyses were performed using STATA 10.1 (StataCorp, College Station, TX, USA).

## Results

### Scan analysis

Mean uptake ratios of counts between the left and right limbs were not different between days 3 and 7 for any of the four groups: WT non-diabetic (*P *= 0.52), WT diabetic (*P *= 0.39), RAGE^-/- ^non-diabetic (*P *= 0.41), and RAGE^-/- ^diabetic (*P *= 0.39). Therefore, days 3 and 7 data were combined as the early time period.

**Figure 1:**
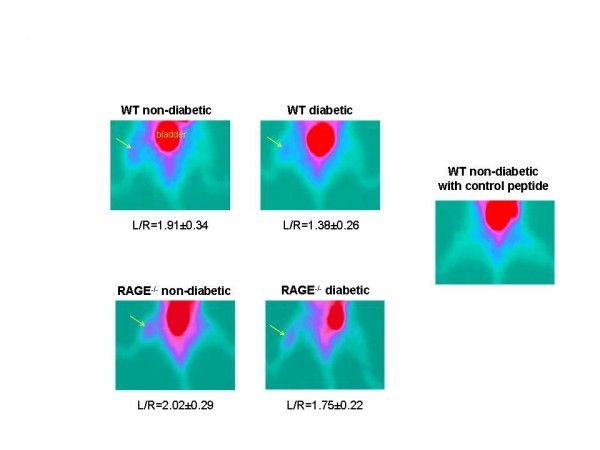
**Representative scans from the four groups and the control peptide**. Images from each of the four groups of mice injected with ^99m^Tc cyclo-RGD and imaged on days 3 to 7 after left femoral artery ligation with mean values for ratios for L/R hindlimb below each image. Image in the right shows a representative scan from an animal injected with control peptide. The yellow arrows point to the tracer uptake. The color table shows the highest counts in red through purple to blue and green is background. The bladder is labeled.

Representative scans from the four groups and the control peptide are shown in Figure 1. All scans in the non-diabetic WT group were positive visually, while three of the left limbs in the diabetic group were negative, one was equivocal, and one weakly positive. Scans of the WT mice injected with control peptide showed no tracer uptake in either limb.

**Figure 2:**
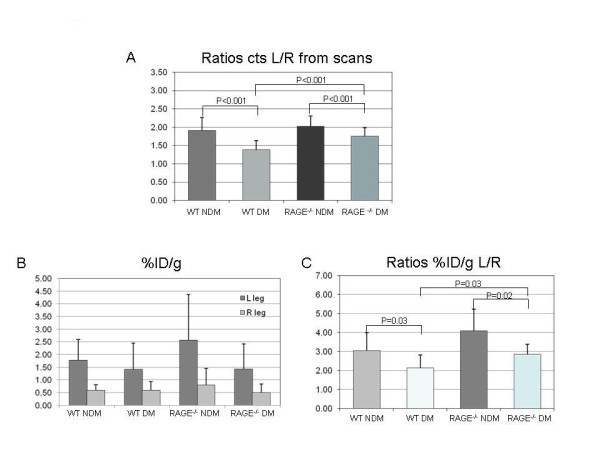
**Data from scans and *ex vivo *well counting for both hindlimbs**. (**A**) Bars represent mean ± standard deviation values for the ratios of left/right (L/R) hindlimbs from the scan count data. (**B**) Bars represent mean ± standard deviation values for %ID/g for both the left and right legs. (**C**) Bars in graph represent mean ± standard deviation values for the ratios for %ID/g for L/R hindlimbs. WT, wild-type; DM, diabetes mellitus; NDM, non-diabetes mellitus.

Data from scans and *ex vivo *well counting for both hindlimbs are shown in Figure 2. For the WT non-diabetic group, the mean scan count ratio for L/R hindlimbs was 1.91 ± 0.34 (range, 1.46 to 2.79), and for the WT diabetic group, it was 1.38 ± 0.26 (range, 1.05 to 1.74) (*P *< 0.001) (Figure 2A). The mean value for the RAGE^-/- ^non-diabetic group was 2.02 ± 0.29 (range, 1.54 to 2.62) not statistically significantly different from the WT non-diabetic group. The mean value for the RAGE^-/- ^diabetic group was 1.75 ± 0.22 (range, 1.53 to 2.35) which was significantly lower than the RAGE^-/- ^non-diabetic group (*P *< 0.001) and was significantly higher than the WT diabetic group (*P *< 0.001).

**Figure 3:**
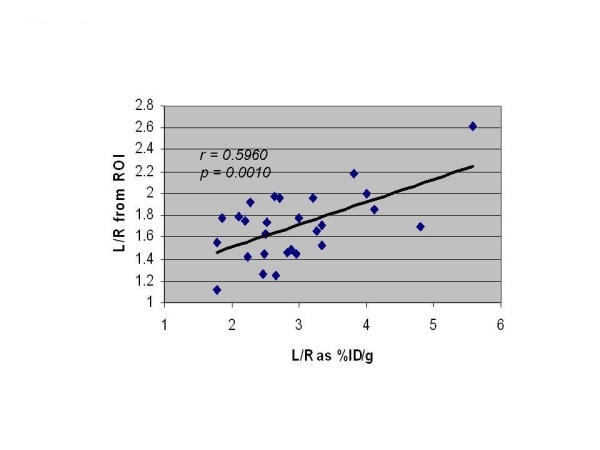
**Correlation of ratio of counts in L/R hindlimb**. From ROIs drawn on the scans correlated with counts from *ex vivo *gamma well counting of the muscles from the left and right hindlimbs and corrected for decay and expressed as %ID/g of tissue.

Figure 2B shows values as %ID/g for the four groups for the left and right hindlimbs. The counts in the left (ischemic) hindlimbs showed the same pattern of differences among the four groups as shown for the scan ratios except for values for the WT diabetic and RAGE^-/- ^diabetic (1.42 and 1.43). However, the ratios of L/R hindlimb %ID/g for RAGE^-/- ^diabetic was higher than for WT diabetic (2.85 ± 0.40 vs. 2.13 ± 0.67, *P *= 0.03) (Figure 2C). This difference is due to lower mean %ID/g in the right limb for RAGE^-/- ^diabetic group. For the remaining limb ratios for %ID/g values, WT non-diabetic was significantly higher than WT diabetic (3.04 ± 0.95 vs. 2.13 ± 0.67, *P *= 0.03) and RAGE^-/- ^non-diabetic was higher than RAGE^-/- ^diabetic (4.08 ± 1.00 vs. 2.85 ± 0.40, *P *= 0.02). All of these significance levels for intergroup differences were lower than for the scan data (Figure 2A) possibly due to the technical challenge to cleanly dissect the anterior tibialis muscles in the mouse. This limitation may have weakened the correlation for the plot of the ratios of L/R limbs against %ID/g (*R *= 0.059), although the correlation is highly significant (*P *= 0.001) (Figure 3).

### Histopathology

**Figure 4:**
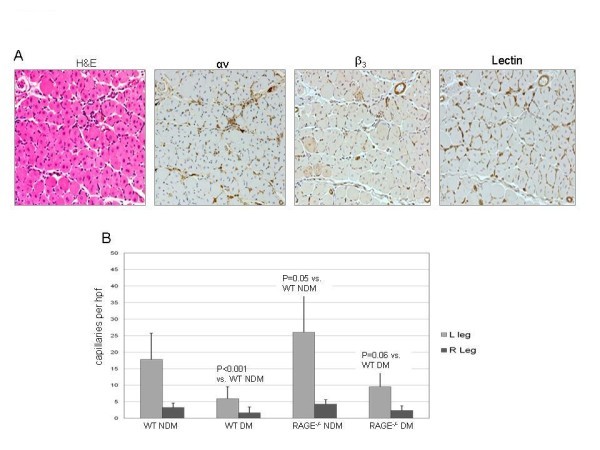
**Tissue sections stained for H&E, α_ν_, β_3_, and lectin**. (**A**) An example of histological and immunohistochemical staining for anterior tibialis muscle sections for a wild-type non-diabetic mouse. (**B**) The bar graph for quantitative lectin staining. Each bar represents average ± SD of lectin-stained capillaries from sections of left anterior tibialis (ischemic limb) (light gray bars) and right anterior tibialis muscle (sham surgery) (dark gray bars) for animals from each of the four groups.

**Figure 5:**
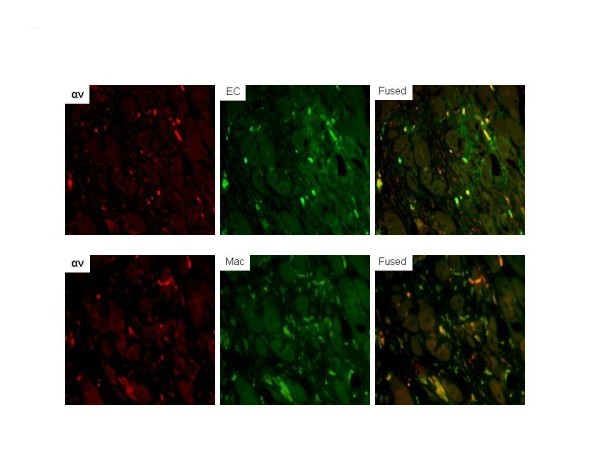
**Dual immunofluorescent staining for cells expressing α_v _in ischemic limb sections**. Sites of α_v _expression were shown to be mainly endothelial cells based on colocalization of α_v _(Texas Red) with FVIII (green, fluorescein isothiocyanate) in the merged image. Colocalization of α_v _with macrophages (Mac-3, fluorescein isothiocyanate) was also seen in the merged image. Areas in yellow represent colocalization. EC, endothelial cells. (Magnification ×200).

Examples of tissue sections stained for H&E, α_ν_, β_3_, and lectin are shown in Figure 4A. Quantitative lectin staining for capillaries from anterior tibialis muscle sections (*n *= 20 per group) for both the left (ischemic) and right (sham operated) hindlimbs of WT non-diabetic, WT diabetic, RAGE^-/- ^non-diabetic, and RAGE^-/- ^diabetic are shown in Figure 4B. The average capillary staining for the WT non-diabetic left limbs was significantly lower than the RAGE^-/- ^non-diabetic left limbs (*P *= 0.05) and significantly higher than the WT diabetic left limbs (*P *< 0.001). The capillary staining for the WT diabetic left limbs was borderline significantly lower than for the RAGE^-/- ^diabetic left limbs (*P *= 0.06). These histological results support the scan findings. Co-staining of sections for both endothelial cells and macrophages showed colocalization with α_ν _(Figure 5).

**Figure 6:**
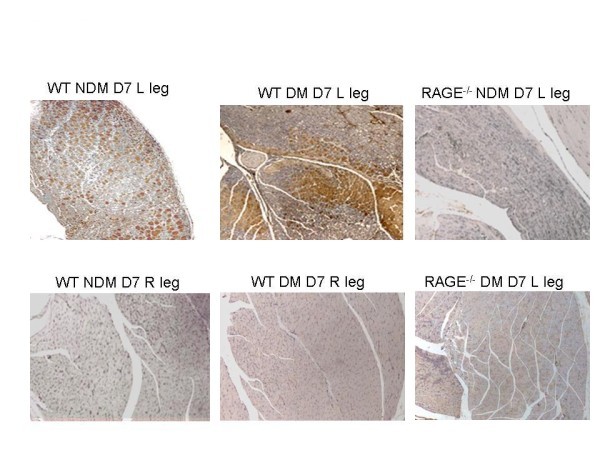
**Representative sections of anterior tibialis muscles stained for RAGE (brown chromagen) and displayed at 20×**. The left set of images shows sections from a left (L) ischemic hindlimb (top) and control right (R) limb (bottom) from a WT non-diabetic (NDM) mouse 7 days (D) after femoral artery ligation. The center set of images shows sections from a left ischemic hindlimb (top) and control right limb (bottom) from a WT diabetic (DM) mouse 7 days after femoral artery ligation. The right set of images shows sections from a left ischemic hindlimb from a RAGE^-/- ^non-diabetic mouse at day 7 after femoral artery ligation (top) and from a RAGE^-/- ^diabetic mouse at day 7 after femoral artery ligation (bottom).

RAGE staining also supported the scan findings. There was positive staining for RAGE in the ischemic sections of hindlimbs from both diabetic and non-diabetic mice, and no staining in the contralateral control limbs (Figure 6). The RAGE^-/- ^mice both non-diabetic and diabetic showed no RAGE staining.

## Discussion

In this study, we used radiolabeled RGD targeting integrin expression and *in vivo *gamma imaging to look at the effects of both diabetes and RAGE expression on the angiogenic response to hindlimb ischemia in mice. By measuring the ratio of tracer uptake in the ischemic limb to the contralateral control limb, we were able to show in live animals that in the absence of RAGE, the angiogenic response to ischemia is ameliorated both in diabetic and non-diabetic mice.

Diabetics have an attenuated angiogenic response to tissue hypoxia which contributes to long-term complications including poor collateral formation in the heart and in the lower extremities which is further aggravated by poor wound healing and ulcers. Several factors have been identified that contribute to this impaired angiogenic response in diabetics which include maladaptive regulation of vascular endothelial growth factor (VEGF) ligand signaling [10-12], impaired release of endothelial progenitor cells from the bone marrow [13], and defective function of the released cells [13,14]. Shoji and co-workers using a matrigel patch model showed that the RAGE system is involved in impaired angiogenesis in diabetes [4].

Under hypoxic conditions, the expression of hypoxia inducible factor (HIF-1) is increased which turns on several genes including genes that code for VEGF that promote angiogenesis to restore perfusion and normoxia in normal subjects. However, exogenous VEGF has no effect to restore blood flow to diabetic mice with limb ischemia and there is reduced downstream VEGF signaling in diabetic animals [10-12]. Tamarat and co-investigators proposed a mechanism involving inhibition of the matrix metalloproteinases (MMPs) proteolytic enzymes that degrade the extracellular matrix, a process that is necessary for the sprouting capillaries as the neovascular mass grows [5]. After 3 days of limb ischemia following femoral artery ligation, MMP-2, MMP-3, and MMP-13 were increased in diabetic mice compared to controls, but collagenolysis was decreased, indicating a suppression of the response. Treatment with aminoguanidine, a thiamine derivative known to inhibit three of the major biochemical pathways in the pathways of angiogenesis including AGE formation, restored the collagenolysis process [5]. Tchaikovski and co-workers investigated mechanisms whereby AGEs and RAGE expression inhibit the response of circulating macrophages and progenitor cells to promote angiogenesis in limb ischemia in diabetes and found activation of VEGFR-1-related signal transduction pathways in monocytes making them resistant to stimulation by VEGF-A [6]. Shen and co-workers showed that both diabetic and non-diabetic mice that received marrow transplantation from RAGE^-/- ^donors had improved limb blood flow at 28 days following ligation compared to mice receiving marrow from RAGE^+/+ ^donors [8].

Integrins are cell adhesion receptors expressed on endothelial cells, and α_v_β_3 _integrin is responsible for cell-cell interaction and the interaction between cells and the extracellular matrix, processes that are necessary for angiogenesis [15-18]. Upon activation of the complex tertiary structure, integrins unfold, revealing a recognition site for the Arg-Gly-Asp (RGD) sequence to bind extracellular matrix (ECM) proteins such as vitronectin, fibrinogen, and fibronectin [19]. This unique peptide binding site was used to develop linear and cyclic peptides with RGD sequence to target α_v_β_3 _integrin for imaging [19,20]. Because α_v_β_3 _integrin is expressed on both endothelial cells and monocyte/macrophages and the inflammatory response to ischemia is increased in diabetes, angiogenesis based on uptake of ^99m^Tc-HYNIC-RGD in the ischemic hindlimbs may have been overestimated in the diabetic mice. Nevertheless, the diabetic mice had significantly lower uptake of ^99m^Tc-HYNIC-RGD in the ischemic limb compared to the non-diabetic mice, suggesting that the binding to endothelial cells in this model had the dominant effect.

Using an RGD mimetic peptide (^99m^Tc-NC100692), Hua and colleagues imaged α_v_β_3 _expression in a murine limb ischemia model [21]. Integrin expression has also been targeted for *in vivo *nuclear imaging in myocardial infarction and remodeling and in response to VEGF therapy in chronic low flow dysfunctional myocardium [22,23]. Our study extends these reports to document the value of this imaging approach to molecular pathways involved in diabetes.

## Conclusions

We confirmed in live animals the role of RAGE expression to inhibit the angiogenic response to limb ischemia in diabetes. Both the diabetic and non-diabetic RAGE^-/- ^mice showed improved angiogenesis compared to the RAGE^+/+ ^mice (WT diabetic and non-diabetic) based on greater uptake of radiolabeled RGD targeting α_v_β_3 _expression, a biomarker of tissue changes accompanying early angiogenesis. While femoral artery occlusion in a mouse is a simple model for limb ischemia compared to the slowly progressive disease in humans, the results of this study support the value of radiolabeled RGD as a non-invasive tool to follow the angiogenic response to modifications in factors affecting the angiogenic response to tissue hypoxia.

## Limitations

Planar imaging was used. As reported in a similar model, planar imaging tends to underestimate relative uptake within lower limbs as compared with SPECT and gamma well counting [21]. Since all experiments were performed the same way, differences among groups are probably not affected; however, such an underestimation would explain the slope of the regression line for the plot of %ID from the scans vs. %ID/g from the tissue.

## Abbreviations

AGEs: advanced glycation endproducts; DM: diabetes mellitus; FA: femoral artery; MMPs: matrix metalloproteinases; NDM: non-diabetes mellitus; %ID/g: percent injected dose per gram; ROI: region of interest; RAGE: receptor for advanced glycation endproducts; VEGF: vascular endothelial growth factor; WT: wild-type.

## Competing interests

The authors declare that they have no competing interests.

## Authors' contributions

YT prepared the tracers, performed the experiments, and revised the manuscript. JL helped in the acquisition of data. DW provided the high-resolution gamma imaging device. AMS and SFY developed the RAGE^-/- ^animal model. RH provided us the RGD peptide. LJ has been involved in designing the experiments, analysis and interpretation of data, and in drafting and revising the manuscript.
